# Supplementary material for: Central nicotine induces browning through hypothalamic κ opioid receptor
Source: Nat Commun. 2019 Sep 6;10:4037. doi: 10.1038/s41467-019-12004-z (PMC6731305; doi:10.1038/s41467-019-12004-z)
Supplement: Supplementary file 1 — Supplementary Information [file 41467_2019_12004_MOESM1_ESM.pdf]

Supplementary Information

Central nicotine induces browning through hypothalamic κ opioid receptor

Patricia Seoane-Collazo *et al*

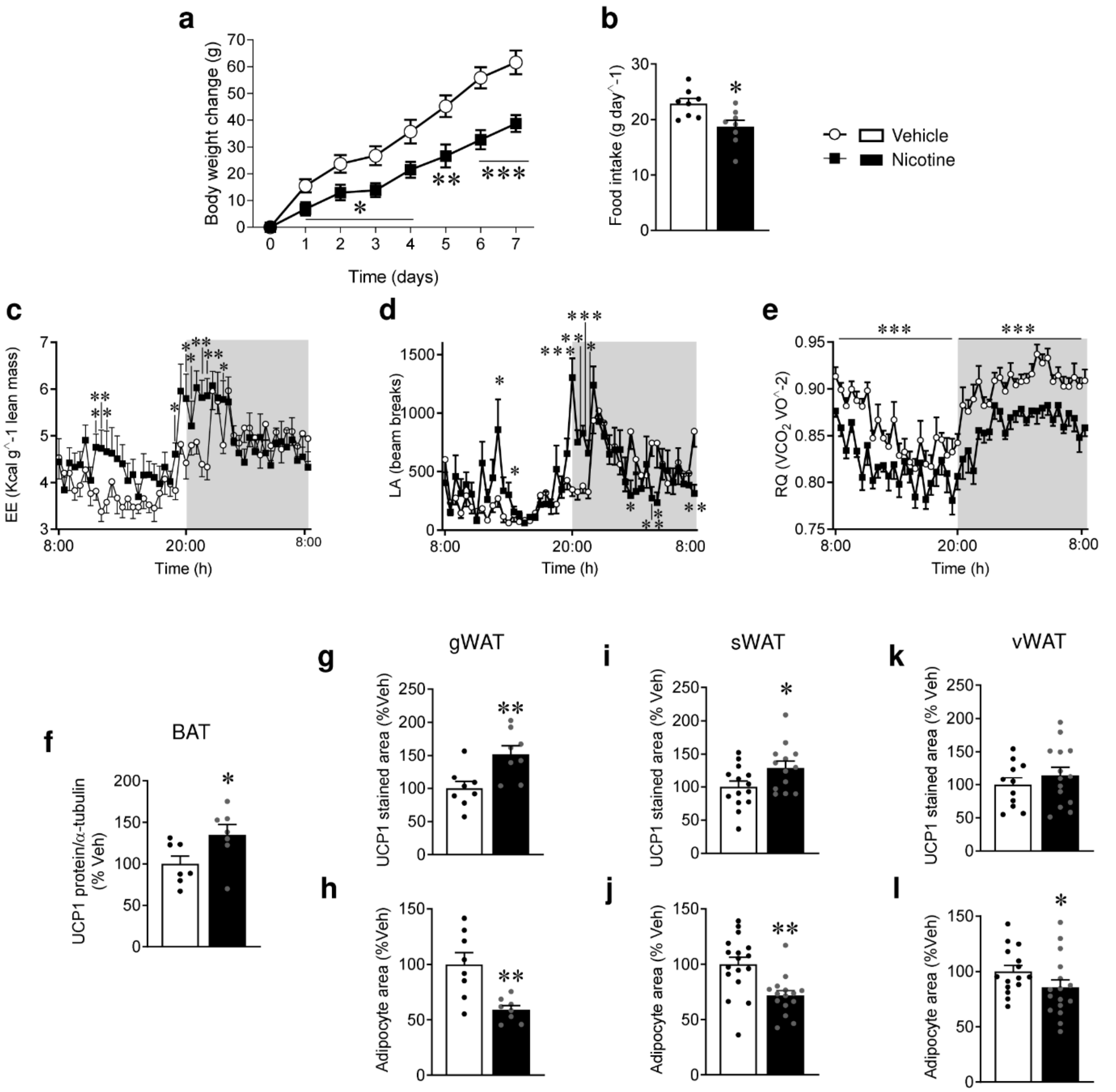

SUPPLEMENTARY FIGURE 1. Effect of peripheral nicotine on energy balance

(a) Body weight change (vehicle n=8, nicotine n=8 rats)
(b) Daily food intake (vehicle n=8, nicotine n=8 rats)
(c) Energy expenditure (vehicle n=13, nicotine n=13 rats)
(d) Locomotor activity (vehicle n=11, nicotine n=11 rats)
(e) Respiratory quotient (vehicle n=13, nicotine n=13 rats)
(f) Protein levels of UCP1 in the BAT (vehicle n=7, nicotine n=7 rats)
(g) UCP1 stained area in gWAT (vehicle n=8, nicotine n=8 rats)
(h) Adipocyte area in gWAT (vehicle n=8, nicotine n=8 rats)
(i) UCP1 stained area in sWAT (vehicle n=14, nicotine n=13 rats)
(j) Adipocyte area in sWAT (vehicle n=17, nicotine n=15 rats)
(k) UCP1 stained area in vWAT (vehicle n=11, nicotine n=14 rats)
(l) Adipocyte area in vWAT (vehicle n=15, nicotine n=16 rats)
of rats SC treated with vehicle or nicotine. Center values represent average, error bars represent SEM. Statistical significance was determined by two-sided t-Student *P<0.05, **P<0.01, ***P<0.001 vs. vehicle. The experiment was repeated three times, the samples represent biological replicates. Source data are provided as a Source Data file.

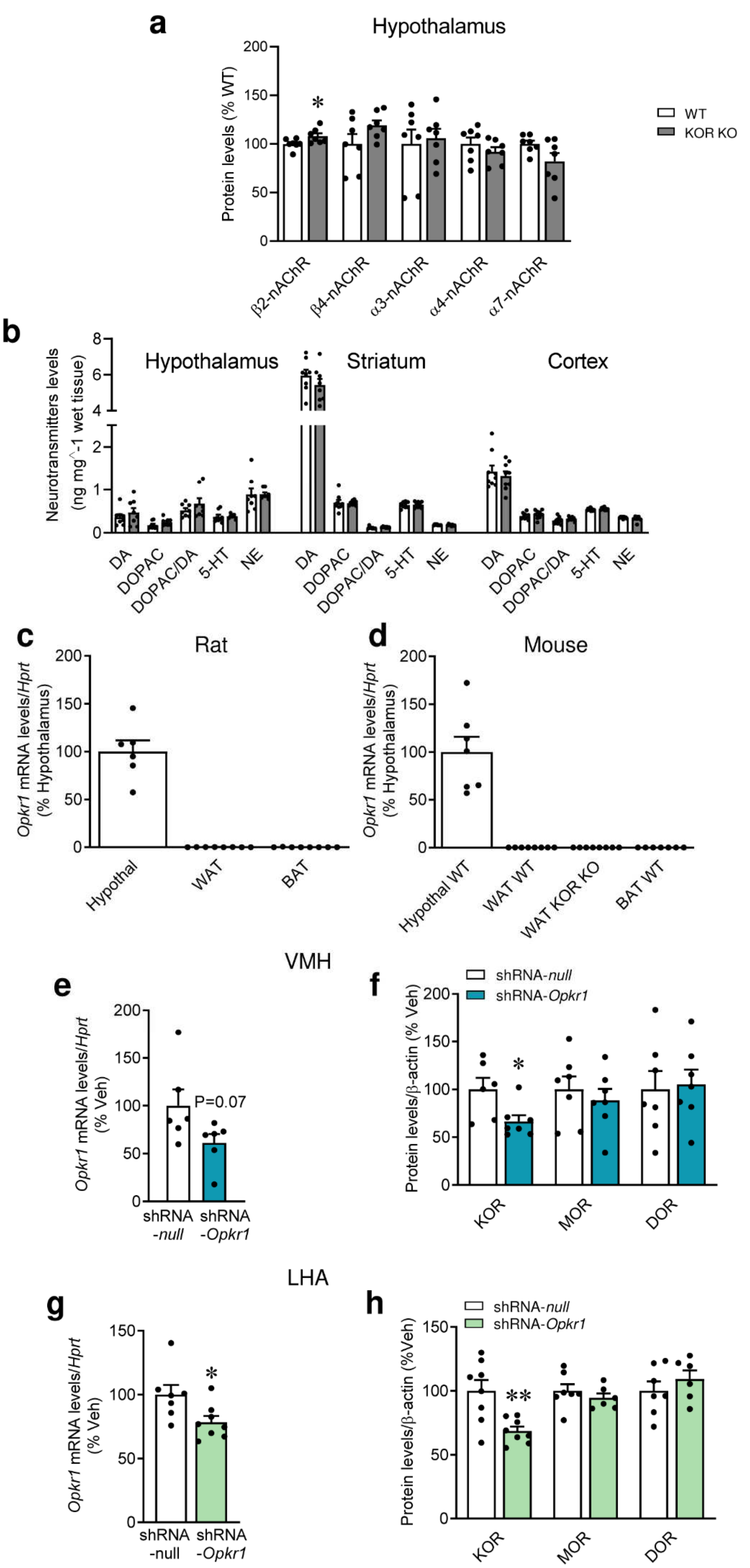
a
Hypothalamus
Protein levels (% WT)
WT
KOR KO
β2-nAChR
β4-nAChR
α3-nAChR
α4-nAChR
α7-nAChR
b
Neurotransmitters levels (ng mg^-1 wet tissue)
Hypothalamus
Striatum
Cortex
DA
DOPAC
DOPAC/DA
5-HT
NE
c
Rat
Opkr1 mRNA levels/Hprt (% Hypothalamus)
Hypothal
WAT
BAT
d
Mouse
Hypothal WT
WAT WT
WAT KOR KO
BAT WT
VMH
e
Opkr1 mRNA levels/Hprt (% Veh)
P=0.07
shRNA -null
shRNA -Opkr1
f
shRNA-null
shRNA-Opkr1
Protein levels/β-actin (% Veh)
KOR
MOR
DOR
LHA
g
Opkr1 mRNA levels/Hprt (% Veh)
shRNA -null
shRNA -Opkr1
h
shRNA-null
shRNA-Opkr1
Protein levels/β-actin (%Veh)
KOR
MOR
DOR

SUPPLEMENTARY FIGURE 2. Molecular characterization of KOR models

(a) Protein levels of cholinergic nicotinic receptors in the hypothalamus of WT and KOR KO mice (WT n=7, KO n=7 mice).

(**b**) Levels of DA, DOPAC, 5-HT and NE in the hypothalamus, striatum and cortex of WT and KOR KO mice (WT n=8, 8, 9, KO n=8, 8, 9 mice). Note that the DOPAC/DA ratio has been expressed in the same graph, even though does not have units.

(c-d) *Opkr1* mRNA levels in hypothalamus, WAT and BAT of rats (hypothalamus n=6, WAT 8, BAT 8 rats) or WT (hypothalamus n=7, WAT n=8, BAT n=7 mice) and KOR KO mice (WAT n=8 mice)

(e) *Opkr1* mRNA levels in VMH (shRNA-*null* n=6, shRNA-*Opkr1* n=6 rats)

(f) Protein levels of KOR, MOR and DOR in the VMH (shRNA-*null* n=6, 7, 7, shRNA-*Opkr1* n=7, 7, 7 rats)

of rats treated with AAVs harboring a shRNA-*null* or a shRNA against *Opkr1* in the VMH.

(g) *Opkr1* mRNA levels in LHA (shRNA-*null* n=7, shRNA-*Opkr1* n=8 rats)

(h) Protein levels of KOR, MOR and DOR levels in LHA (shRNA-*nul*l n=8, 7, 7, shRNA-*Opkr1* n=8, 6, 6 rats)

of rats treated with AAVs harboring a shRNA-*null* or a shRNA against *Opkr1* in the LHA. Center values represent average error bars represent SEM. Statistical significance was determined by two-sided t-Student *P<0.05, **P<0.01 vs. shRNA-*null.* Source data are provided as a Source Data file.

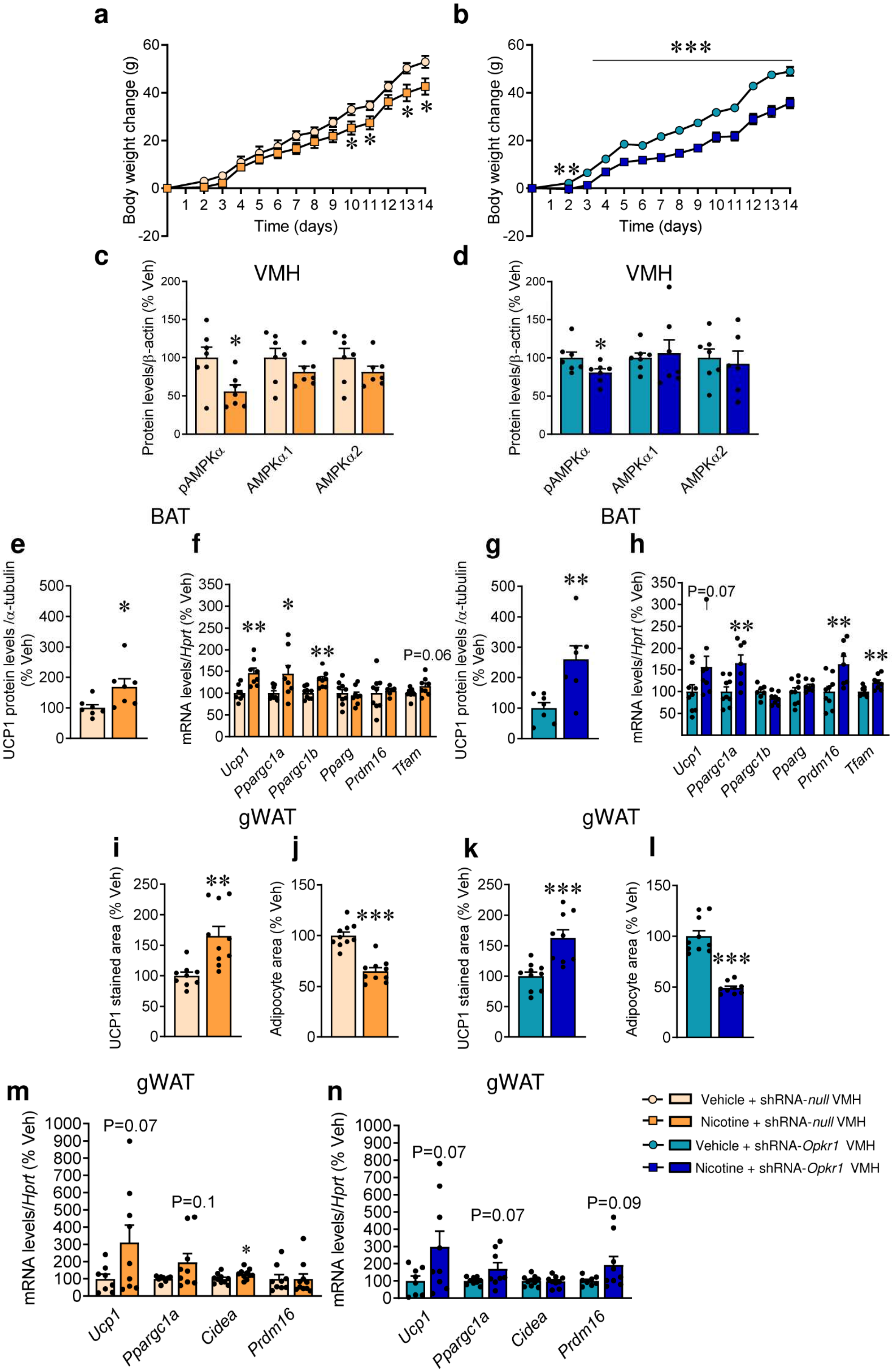

a
b
Body weight change (g)
Time (days)
c
d
VMH
Protein levels/β-actin (% Veh)
pAMPKα
AMPKα1
AMPKα2
BAT
e
f
g
h
UCP1 protein levels /α-tubulin (% Veh)
mRNA levels/Hprt (% Veh)
Ucp1
Ppargc1a
Ppargc1b
Pparg
Prdm16
Tfam
P=0.06
P=0.07
gWAT
i
j
k
l
UCP1 stained area (% Veh)
Adipocyte area (% Veh)
m
n
Cidea
P=0.1
P=0.09
Vehicle + shRNA-null VMH
Nicotine + shRNA-null VMH
Vehicle + shRNA-Opkr1 VMH
Nicotine + shRNA-Opkr1 VMH

SUPPLEMENTARY FIGURE 3. Effect of VMH *Opkr1* knockdown on nicotine's effect on BAT and WAT

(a-b) Body weight (vehicle + shRNA-*null* n=10, nicotine + shRNA-*null* n=10, vehicle + shRNA-*Opkr1* n=10, nicotine + shRNA-*Opkr1* n=9 rats)
(c-d) Protein levels of AMPK in the VMH (vehicle + shRNA-*null* n=7, nicotine + shRNA-*null* n=7, vehicle + shRNA-*Opkr1* n=7, nicotine + shRNA-*Opkr1* n=7, 7, 6 rats)
(e, g) Protein levels of UCP1 in the BAT (vehicle + shRNA-*null* n=7, nicotine + shRNA-*null* n=7, vehicle + shRNA-*Opkr1* n=7, nicotine + shRNA-*Opkr1* n=7 rats)
(f, h) mRNA levels of thermogenic markers in the BAT (vehicle + shRNA-*null* n=8, 8, 9, 10, 9, 9, nicotine + shRNA-*null* n=8, 8, 8, 8, 6, 8, vehicle + shRNA-*Opkr1* n=9, 9, 7, 9, 9, 9, nicotine + shRNA-*Opkr1* n=8, 7, 8, 7, 7, 8 rats)
(i, k) UCP1 stained area in WAT (vehicle + shRNA-*null* n=9, nicotine + shRNA-*null* n=10, vehicle + shRNA-*Opkr1* n=10, nicotine + shRNA-*Opkr1* n=9 rats)
(j, l) Adipocyte area in WAT (vehicle + shRNA-*null* n=10, nicotine + shRNA-*null* n=10, vehicle + shRNA-*Opkr1* n=10, nicotine + shRNA-*Opkr1* n=9 rats)
(m-n) mRNA levels of thermogenic markers in the WAT (vehicle + shRNA-*null* n=8, 8, 10, 9, nicotine + shRNA-*null* n=9, 9, 10, 10, vehicle + shRNA-*Opkr1* n=8, 9, 10, 8, nicotine + shRNA-*Opkr1* n=9, 8, 9, 9 rats)
of rats stereotaxically treated within the VMH with AAVs harboring a shRNA-*null* (**a, c, e, f, i, j and m**) or a shRNA against *Opkr1* (**b, d, g, h, k, l and n**) and treated with vehicle or nicotine. Center values represent average error bars represent SEM. Statistical significance was determined by two-sided t-Student *P<0.05, **P<0.01, ***P<0.001 vs. vehicle. Source data are provided as a Source Data file.

SUPPLEMENTARY TABLE 1. Anthropometric and clinical parameters of participants from both cohort 1 and 2

**Cohort 1**	**No smoker**	**Current smoker**	**P-value**
N	20	5	
Sex (men/women)	3/17	2/3	
Age (years)	39.05 ± 8.9	39.80 ± 6.14	0.8
BMI (kg m^{-2})	36.35 ± 9.39	38.50 ± 7.30	0.6
Fasting glucose (mg dl^{-1})	93.5 (80.2-102)	96 (87-131.5)	0.3
Total cholesterol (mg dl^{-1})	194.21 ± 24.2	210.80 ± 30.78	0.2
HDL-cholesterol (mg dl^{-1})	57.20 ± 11.55	63.75 ± 11.08	0.3
LDL- cholesterol (mg dl^{-1})	114.66 ± 27.56	130.75 ± 42.50	0.3
Fasting triglycerides (mg dl^{-1})	93.5 (61.7-160.7)	114 (69-225)	0.4
**Cohort 2**	**No smoker**	**Current smoker**	**P-value**
N	40	16	
Sex (men/women)	11/29	5/11	
Age (years)	46 ± 5 8.1	48.3 ± 8.8	0.5
BMI (kg m^{-2})	36.6 ± 11.7	34.5 ± 12.8	0.5
Fasting glucose (mg dl^{-1})	100.9 ± 28.2	93.6 ± 15.3	0.3
Total cholesterol (mg dl^{-1})	198.9 ± 36.1	210.9 ± 46.3	0.3
HDL-cholesterol (mg dl^{-1})	52.8 ± 14.8	45.5 ± 13.8	0.1
LDL- cholesterol (mg dl^{-1})	124.2 ± 33.9	139.1 ± 34.9	0.2
Fasting triglycerides (mg dl^{-1})	109.1 ± 51.8	131.2 ± 69.7	0.2
Serum Cotinine (ng ml^{-1})	0	258.4 ± 24.5	<0.0001

Mean ± standard deviation for normal distributed variables and median (interquartile range) for non-normal distributed variables. Qualitative variables are expressed as frequencies. BMI, body mass index; HDL, high density lipoprotein; LDL, low density lipoprotein. Statistical analysis was performed using one factor ANOVA with Bonferroni post hoc test.

SUPPLEMENTARY TABLE 2. Multivariate regression analyses to predict vWAT UCP1 gene expression in participants from cohort 2 (N= 56)

vWAT UCP1 (RU)*	Model 1		Model 2		Model 3	
	**β**	**P**	**β**	**p**	**β**	**p**
Age (years)	0.09	0.5	-	-	0.06	0.6
BMI (kg m^{-2})	-	-	-0.21	0.1	-0.19	0.1
Serum cotinine (ng ml^{-1})	0.28	0.03	0.28	0.03	0.27	0.04
Adjusted R^2	0.058 (5.8%)		0.093 (9.3%)		0.079 (7.9%)	
P (model)	0.07		0.02		0.06	

SUPPLEMENTARY TABLE 3. Primers and probes for real-time PCR *(TaqMan®)* analysis in rodents

mRNA	GenBank Accession Number		Sequence
Cidea (rat)		Assay ID	ThermoFisher TaqMan® Gene Expression Assays<br>Assay ID Rn04181355_m1
Cidea (mouse)	NM_007702.2	Fw Primer	5'-CCTACGACATCCGATGCACAA-3'
		Rv Primer	5'-TCTGTGCAGCATAGGACATAAACC-3'
		Probe	FAM-5'-CTTCAAGGCCGTGTTAAGGAATCTGCTG-3'-TAMRA
Hprt	NM_012583	Fw Primer	5'-AGCCGACCGGTTCTGTCAT-3'
		Rv Primer	5'-GGTCATAACCTGGTTCATCATCAC-3'
		Probe	FAM-5'-CGACCCTCAGTCCCAGCGTCGTGAT-3'-TAMRA
Ppargc1a	NM_031347	Fw Primer	5'-CGATCACCATATTCCAGGTCAAG-3'
		Rv Primer	5'-CGATGTGTGCGGTGTCTGTAGT-3'
		Probe	FAM-5'-AGGTCCCCAGGCAGTAGATCCTCTTCAAGA-3'-TAMRA
Ppargc1b		Assay ID	Applied Biosystems TaqMan® Gene Expression Assays<br>Assay ID Rn00598552_m1
Prdm16		Assay ID	ThermoFisher TaqMan® Gene Expression Assays<br>Assay ID Mm01266512_m1
Ucp1 (rat)	NM_012682	Fw Primer	5'-CAATGACCATGTACACCAAGGA-3'
		Rv Primer	5'-GATCCGAGTCGCAGAAAAGAA-3'
		Probe	FAM-5'-ACCGGCAGCCTTTTTCAAAGGGTTTG-3'-TAMRA

Ucp1 (mouse)	NM_009463	Fw Primer<br>Rv Primer<br>Probe	5'-CAATGACCATGTACACCAAGGAA-3'<br>5'-GACCCGAGTCGCAGAAAAGAA-3'<br>FAM-5’-ACCGGCAGCCTTTTTCAAAGGGTTTG-3'-TAMRA
Tfam		Assay ID	ThermoFisher TaqMan® Gene Expression Assays<br>Assay ID Rn00580051_m1
