# Supplementary material for: Central nicotine induces browning through hypothalamic κ opioid receptor
Source: Nat Commun. 2019 Sep 6;10:4037. doi: 10.1038/s41467-019-12004-z (PMC6731305; doi:10.1038/s41467-019-12004-z)
Supplement: Supplementary file 3 — Description of Additional Supplementary Files [file 41467_2019_12004_MOESM3_ESM.pdf]

Description of Additional Supplementary Files

File name: Supplementary Movie 1

Description: Effect of nicotine on the sympathetic fibers innervating gWAT. Tyroxine hydroxylase (TH) immunostaining coupled to Adipo-Clear of gWAT fat pads of mice ICV-treated with vehicle (left) and nicotine (right).
