# Supplementary material for: Central nicotine induces browning through hypothalamic κ opioid receptor
Source: Nat Commun. 2019 Sep 6;10:4037. doi: 10.1038/s41467-019-12004-z (PMC6731305; doi:10.1038/s41467-019-12004-z)
Supplement: Supplementary file 5 — Reporting Summary [file 41467_2019_12004_MOESM5_ESM.pdf]

nature research

Corresponding author(s): Carlos Diéguez & Miguel López

Last updated by author(s): Aug 9, 2019

Reporting Summary

Nature Research wishes to improve the reproducibility of the work that we publish. This form provides structure for consistency and transparency in reporting. For further information on Nature Research policies, see Authors & Referees and the Editorial Policy Checklist.

Statistics

For all statistical analyses, confirm that the following items are present in the figure legend, table legend, main text, or Methods section.

n/a	Confirmed	
☐	☒	The exact sample size (*n*) for each experimental group/condition, given as a discrete number and unit of measurement
☐	☒	A statement on whether measurements were taken from distinct samples or whether the same sample was measured repeatedly
☐	☒	The statistical test(s) used AND whether they are one- or two-sided<br>*Only common tests should be described solely by name; describe more complex techniques in the Methods section.*
☐	☒	A description of all covariates tested
☒	☐	A description of any assumptions or corrections, such as tests of normality and adjustment for multiple comparisons
☐	☒	A full description of the statistical parameters including central tendency (e.g. means) or other basic estimates (e.g. regression coefficient) AND variation (e.g. standard deviation) or associated estimates of uncertainty (e.g. confidence intervals)
☐	☒	For null hypothesis testing, the test statistic (e.g. *F*, *t*, *r*) with confidence intervals, effect sizes, degrees of freedom and *P* value noted<br>*Give P values as exact values whenever suitable.*
☒	☐	For Bayesian analysis, information on the choice of priors and Markov chain Monte Carlo settings
☒	☐	For hierarchical and complex designs, identification of the appropriate level for tests and full reporting of outcomes
☐	☒	Estimates of effect sizes (e.g. Cohen's *d*, Pearson's *r*), indicating how they were calculated

Our web collection on statistics for biologists contains articles on many of the points above.

Software and code

Policy information about availability of computer code

Data collection	ImageJ 1.44p; GrahPad Prism 8; Shimadzu LC Solution Software; Skyscan's NRecon Software; IMARIS 9.2 Software
Data analysis	Microsoft Excel 365; ImageJ 1.44p; GrahPad Prism 8; Shimadzu LC Solution Software; Skyscan's NRecon Software; IMARIS 9.2 Software; SPSS 12.0 Software

For manuscripts utilizing custom algorithms or software that are central to the research but not yet described in published literature, software must be made available to editors/reviewers. We strongly encourage code deposition in a community repository (e.g. GitHub). See the Nature Research guidelines for submitting code & software for further information.

Data

Policy information about availability of data

All manuscripts must include a data availability statement. This statement should provide the following information, where applicable:

- Accession codes, unique identifiers, or web links for publicly available datasets
- A list of figures that have associated raw data
- A description of any restrictions on data availability

The data that support the findings of this study are available from the corresponding author upon reasonable request. The source data underlying Figures and Supplementary Figures are provided as a Source Data file.

Field-specific reporting

Please select the one below that is the best fit for your research. If you are not sure, read the appropriate sections before making your selection.

☒ Life sciences ☐ Behavioural & social sciences ☐ Ecological, evolutionary & environmental sciences

For a reference copy of the document with all sections, see nature.com/documents/nr-reporting-summary-flat.pdf

Life sciences study design

All studies must disclose on these points even when the disclosure is negative.

Sample size	For animal experiments, sample size was chosen based on similar previous studies of our group and on the basis of literature documentation of similar well characterized experiments. We try to use the fewest number of mice to achieve statistical significance without compromising the outcomes.<br><br>For the human studies, two independent cohorts (cohort 1, n=25 and cohort 2, n=56) were studied according to smoking status. All these subjects were recruited at the Endocrinology Service of the Hospital of Girona “Dr Josep Trueta”, were of Caucasian origin and reported that their body weight had been stable for at least three months before the study. Subjects were studied in the post-absorptive state. Patients had no systemic disease other than obesity and all were free of any infections in the previous month before the study. Liver diseases (specifically tumoral disease and HCV infection) and thyroid dysfunction were specifically excluded by biochemical work-up.
Data exclusions	Samples were excluded whether their values were outside the 2SD range. Criteria were pre-established.
Replication	All attempts to replicate the experiments have been successful. Experiments were repated at least twice.
Randomization	Before starting an experiment, all animal groups were made with set of animals of the same sex, age and similar body weight. Anthropometric parameters for human studies are specified in Supplementary Table 1.
Blinding	For practical reasons, the investigators were not blinded to allocation during experiments and outcome assessment.

Reporting for specific materials, systems and methods

We require information from authors about some types of materials, experimental systems and methods used in many studies. Here, indicate whether each material, system or method listed is relevant to your study. If you are not sure if a list item applies to your research, read the appropriate section before selecting a response.

Materials & experimental systems

n/a	Involved in the study
☐	☒ Antibodies
☒	☐ Eukaryotic cell lines
☒	☐ Palaeontology
☐	☒ Animals and other organisms
☐	☒ Human research participants
☒	☐ Clinical data

Methods

n/a	Involved in the study
☒	☐ ChIP-seq
☒	☐ Flow cytometry
☒	☐ MRI-based neuroimaging

Antibodies

Antibodies used	UCP1 (1:10000; ab10983), MOR (1:1000; ab17934), β2-nAChR (1:5000; ab41174), β4-nAChR (1:1000; ab129276), α2α3-nAChR (1:1000; ab183097), α4-nAChR (1:1000; ab41172), α7-nAChR (1:1000; ab216485) (Abcam; Cambridge, UK); β-actin (1:5000; A5316), α-tubulin (1:5000; T5168), KOR (1:1000; SAB2501442), DOR (1:1000; SAB4502042) (Sigma; St Louis, MO, USA); AMPKα1 (1:1000; 07-350), AMPKα2 (1:1000; 07-363) (Millipore; Billerica, MA, USA), pAMPKα-Thr172 (1:1000; 2535S) (Cell Signaling; Danvers; MA, USA); TH (1:500; ab152; Merk Millipore; Burlington, MA, US). All details provided in the Methods and in Excel Data Source Files.
Validation	All antibodies used have been validated by the manufacturer and by references, including our former papers.

Animals and other organisms

Policy information about studies involving animals; ARRIVE guidelines recommended for reporting animal research

Laboratory animals	Adult male Sprague-Dawley rats (8-10 weeks old, 200-250 g; Animalario General USC, Santiago de Compostela, Spain); adult male null κ opioid receptor (KOR KO) mice and their wildtype controls and C57BL/6J mice (8-12 weeks old, 20-25g) were used.

Wild animals	n/a
Field-collected samples	The animals were housed with a 12-hour light (8:00 to 20:00)/12-hour dark cycle, under controlled temperature and humidity conditions and allowed free access to standard laboratory chow (STD, SAFE A04: 3,1% fat, 59,9% carbohydrates, 16,1% proteins, 2.791 kcal/g; Scientific Animal Food & Engineering; Nantes, France) and tap water.
Ethics oversight	All experiments were performed in agreement with the International Law on Animal Experimentation and were approved by the USC Ethical Committee (Project ID 15010/14/006), Gulbenkian Ethics Committee (Project ID A011.2016) and the University of Iowa Institutional Animal Care and Use Committee.

Note that full information on the approval of the study protocol must also be provided in the manuscript.

Human research participants

Policy information about studies involving human research participants

Population characteristics	Two independent cohorts (cohort 1, n=25 and cohort 2, n=56) were studied according to smoking status. Anthropometric (sex, age and BMI) and clinical (circulating levels of glucose, total cholesterol, HDL cholesterol, LDL cholesterol and triglycerides, as well as cotinine) parameters from these participants are detailed in Supplementary Table 1.
Recruitment	Patients had no systemic disease other than obesity and all were free of any infections in the previous month before the study. Liver diseases (specifically tumoral disease and HCV infection) and thyroid dysfunction were specifically excluded by biochemical work-up. Adipose tissue samples were obtained during elective surgical procedures (cholecystectomy, surgery of abdominal hernia and gastric by-pass surgery).
Ethics oversight	Samples and data from patients included in this study were partially provided by the FATBANK platform promoted by the CIBEROBN and coordinated by the IDIBGI Biobank (Biobank IDIBGI, B.0000872), integrated in the Spanish National Biobanks Network and they were processed following standard operating procedures with the appropriate approval of the Ethics, External Scientific and FATBANK Internal Scientific Committees.

Note that full information on the approval of the study protocol must also be provided in the manuscript.
